# Co-teaching in higher education: implications for teaching, learning, engagement, and satisfaction

**DOI:** 10.3389/fspor.2024.1424101

**Published:** 2024-07-22

**Authors:** Sima Zach, Simcha Avugos

**Affiliations:** Levinsky-Wingate Academic College, Wingate Campus, Netanya, Israel

**Keywords:** co-teaching, teacher education, student engagement, teaching satisfaction, teachers’ well-being

## Abstract

**Introduction:**

This study examined the impact of co-teaching on students and lecturers, assessing its benefits and drawbacks, and suggesting ways to enhance collaborative learning.

**Methods:**

Fifty undergraduate student teachers participated in two sports sciences seminar courses jointly taught by two lecturers. Data was collected via student reflections; course evaluation feedback; word clouds; and teacher reflections. Thematic analysis was used for qualitative data.

**Results:**

The findings indicate that the short intensive seminar course resulted in three parallel processes: *emotional*, students transitioning from negative feelings of chaos, frustration, and a sense of incompetence to positive feelings of satisfaction and sense of accomplishment; *social*, students learning to listen, request assistance, support, encourage, and collaborate; and *cognitive*, students learning to ask fruitful questions, plan experiments, summarize, and present. Nevertheless, the time and effort demands involved in the planning and management of such courses may constitute a significant barrier to the future implementation of this teaching method. In terms of course outcomes, no indications of higher quality were seen compared to traditional instruction.

**Discussion:**

Drawing on the symbolic interactionism theory, the study advocates for preparing students for inclusive and collaborative learning environments to improve academic engagement and success.

## Introduction

In recent years, the higher education landscape has undergone significant changes, warranting a fundamental shift in instructional approaches. In response, a range of innovative pedagogical methods have emerged, such as co-teaching (1), team-teaching (2), peer teaching (3, 4), and multi-disciplinary and inter-disciplinary teaching (5). This study, guided by insights from the social theory of symbolic interactionism, aims at exploring the significance of integrating co-teaching practices in higher education, specifically within teacher education.

**Figure 1:**
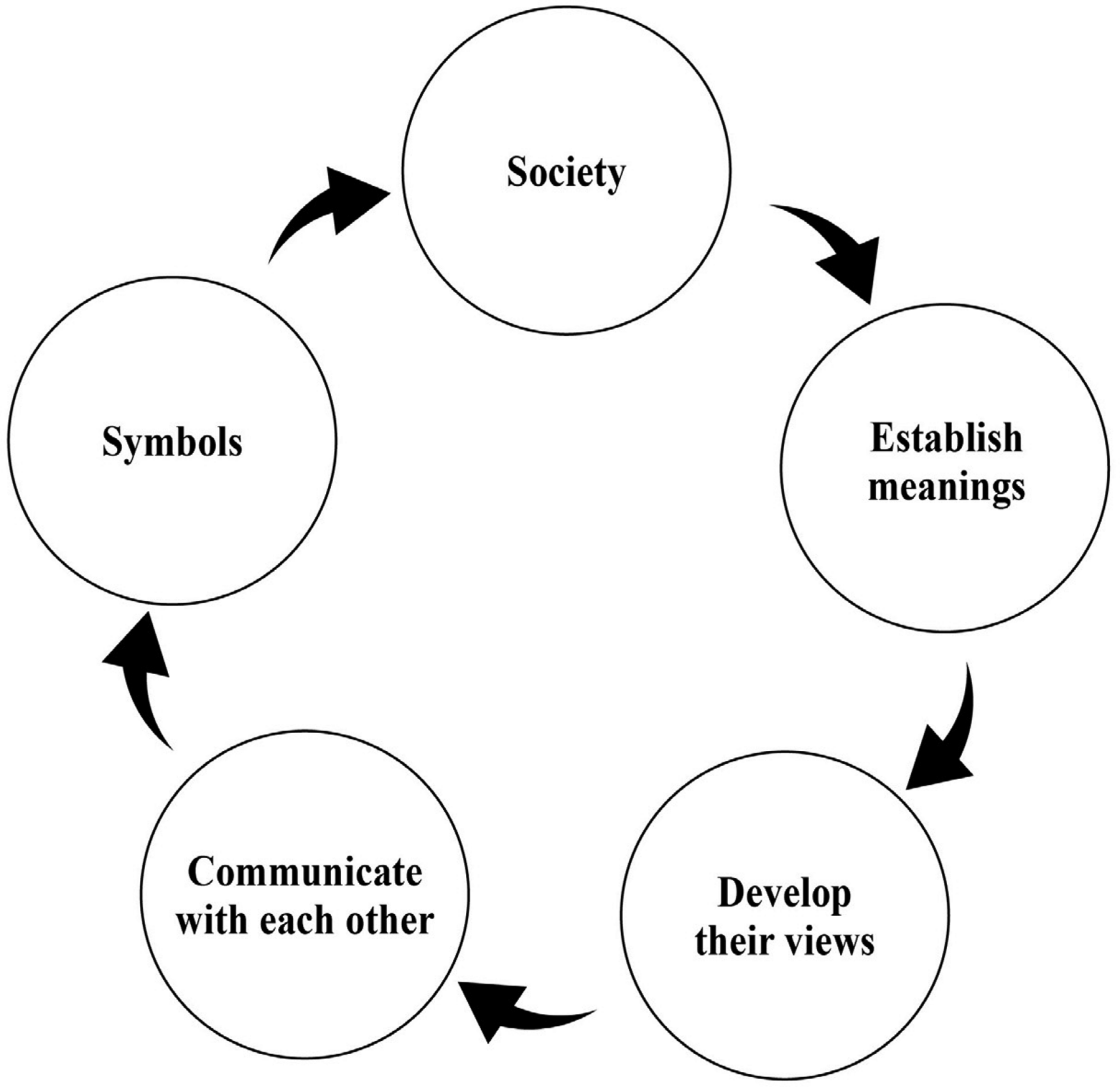
The symbolic interactionism theoretical framework.

Symbolic interactionism asserts that individuals construct meaning through interactions with others and with their environment (6), as depicted in Figure 1. Co-teaching is an instructional approach that involves two or more educators who collaborate to provide students with instruction and support in a shared classroom (7). Originally rooted in special education, as a means for addressing the diverse needs of the students, co-teaching has gained traction in higher education, in light of its potential to improve learning outcomes, enhance student engagement, and foster the professional development of academic staff (8, 9). By harnessing the combined expertise of instructors, co-teaching creates a supportive and inclusive learning environment, catering to the diverse needs of students while maximizing their learning potential (7).

The co-teaching concept first emerged in the 1970s following the diversity and inclusion movement in the United States, which strove to ensure the integration of students with disabilities into general education classrooms (10). Initially designed to address the diverse learning needs of such students, co-teaching entailed collaboration between teachers of special education and of general education. Over time, this approach expanded beyond special education settings, becoming an acceptable form of teaching in higher education as a means for accommodating the increasing diversity of the students and their learning styles (11). In higher education, co-teaching fosters a collaborative learning environment by bringing together diverse perspectives and expertise. The synergy between co-teachers encourages open dialogue, active learning, and exploration of concepts from multiple vantage points, while promoting critical thinking and preparing students for collaborative workforces (12, 13).

The benefits of co-teaching in higher education are numerous. First, it enhances student learning by incorporating a variety of teaching styles and instructional methods, leading to improved learning outcomes (14, 15). In addition, such co-teaching increases student engagement through active participation, peer interaction, and dynamic discussions, thereby fostering social, motivational, and cognitive dimensions of engagement (16). Moreover, it exposes students to multiple teaching approaches, while encouraging active learning and enhancing their understanding and retention of course materials. It creates an interactive and student-centered learning environment—one that motivates students to actively participate in the learning process. The fourth advantage of co-teaching can be seen in its facilitating of individualized support, while enabling the educators to address their students' diverse needs (17). This allows for tailored support and ensures that every student receives adequate and suitable attention and assistance. Additionally, co-teaching promotes collaboration and professional growth among academic staff, enabling them to share their expertise, learn from each other, and develop pedagogical practices (18). Doing so fosters a culture of continuous improvement, as educators are actively engaged in collaborative reflection and in exploring innovative teaching methodologies. Finally, co-teaching decreases teachers’ feelings of isolation and loneliness, through shared responsibilities, workloads, planning, and assessments—which could lead to a more sustainable teaching environment (19).

However, co-teaching is not without certain shortcomings. First, it requires additional time and resources for planning, coordinating, and communicating, which can be challenging in busy academic environments (20). Moreover, ambiguity in the defining and execution of roles and responsibilities may lead to confusion among one of both teachers, potentially affecting the instructional efficiency (21). Additionally, differences in teaching styles and approaches among co-teachers may hinder effective collaboration, requiring additional time and effort for aligning methodologies (22). Finally, co-teaching may not be feasible in all institutions, especially those with limited human resources and budgets (23).

To address these challenges and enable positive change, the current research proposes a personal and pedagogical shift in teaching and learning using an action research method. The focus is on understanding how students learn and how course curricula can be designed, to better address the learners' cognitive, social, and emotional needs. The study strives to trigger changes in both the teachers-researchers and their students, ultimately leading to the modifying and updating of existing teacher training programs. The systematic reflective investigation of teaching-learning processes, as applied in this study, is expected to develop awareness, capabilities, commitment, and engagement among the participating teacher-researchers. In turn, this may facilitate positive transformations in the learning experiences of both educators and student teachers.

It is important to note that there is significant discrepancy between the theories of adult education and the actual practices implemented in the field. Particularly within academic instruction, the emphasis tends to be on high levels of discipline-based teaching, with a focus on the future teacher acquiring expertise in the subject matter (24, 25). Unfortunately, this often leads to the neglecting of the socio-emotional needs of the learner (i.e., the future teacher), such as active learning, collaboration, feedback, and efficient reflection. The choice made by lecturers in academia to prioritize these aspects in instruction is not always guaranteed. Hence, the current study aims at addressing the following three objectives: (1) Examining the impact of co-teaching on students and their lecturers' satisfaction, thoughts, and feelings, using problem-based-learning (PBL) and cooperative learning models; (2) Exploring perceptions of the advantages and shortcomings of the co-teaching strategy, as perceived by both students and lecturers; and (3) Evaluating the efficacy, accountability, and involvement of students in team-based learning, while gathering suggestions for improving team learning. Moreover, by investigating the influence of co-teaching and its impact on both students and lecturers, this study seeks to bridge the gap between theoretical concepts and practical applications in the field of adult education for the benefit of students training to become teachers of tomorrow and their future students.

## Method

### Participants

The study included 50 undergraduate student teachers (26 females) aged 22–36 (*M* = 26.57, SD = 2.31) who were registered for one of two parallel seminar courses in the field of sports sciences. Each seminar consisted of 12 lessons, each lasting three academic hours, over a 3-week period during the summer semester. For about two-thirds of the students, the seminar was their first academic research project. All students were in the third or fourth year of their Bachelor of Education (B.Ed.) studies in sports education at a college of education in the center of Israel.

### Research design and tools

The study was conducted based on the action research approach, with a focus on the deliberate and critical self-reflection as a means for improving our pedagogy and practice as educators, while enhancing the students' engagement and learning. To this end, the following tools were applied for data collection: (1) student reflections of their experiences during the course; (2) course evaluation feedback; (3) cloud of words; and (4) teacher reflections.

To gather data through *student reflections*, at the end of the second and fifth lessons, the students were asked to submit free-writing reflections of their thoughts and feelings regarding their experiences on the course. This included detailed descriptions of the processes that they underwent when preparing their research projects, with an emphasis on the benefits of working together in a small group, the manner in which they supported their teammates during the research process, and the contribution of their teammates to their individual progress. Additionally, they were asked to write their opinion on how their group work could be improved, and what they needed for achieving better outcomes in their projects.

Data was also gathered through *course evaluation feedback.* During the final lesson of each course, the students completed a paper-and-pencil questionnaire that included open-ended questions. They were asked to share their views of the course, including positive aspects and areas that needed improvement. The students were also asked to express their personal preferences towards a course that is jointly taught by two lecturers, using PBL in small groups (as experienced in the current course), or the traditional method of a single teacher per class—and to explain their choice. The questionnaire also included five Likert-type questions. The questions covered topics such as the learning that they achieved through presentation assignments, the efforts that they invested in the course, and their learning processes throughout the research project.

Finally, at the end of the course, each student was asked to write one word that came to mind in the context of the course, i.e., *cloud of words*. Both teachers were also asked to submit a *teacher reflection*, based on free and spontaneous writing, without re-reading or editing what they had written, even in cases of repetition. In these writing, they were asked to reflect on their actions and experiences during the course.

### Procedure

Traditionally, the students on this seminar course were divided into two discrete groups, each with a different lecturer. In the summer of 2023, a new course format was introduced, initiated by the lecturers of the two courses. This innovative approach involved collaborative teaching (co-teaching), where the two teachers jointly instructed and mentored the same group of students in a shared space. The students worked in small groups on various learning tasks. The experiences and outcomes of this collaborative teaching approach, from both the students' and the teachers' perspective, are presented in this paper.

Prior to participating in the study, the students were informed about the research goals and provided their informed written consent. Anonymity was ensured to all participants; moreover, they were informed that their feedback regarding the course for the purpose of this study would not impact their course grade. The study was approved by the college's Institutional Review Board (IRB approval # 255).

The course structure and content were prepared in advance, including a conceptual and practical framework, yet with certain degrees of flexibility—to enable the adapting of the content and tasks, as per the students' progress with their research projects. During the first lesson, the students were divided into working groups, each with four students—two from each original course group. The aim was to encourage dialogue between students from different yet related courses, as a means for fostering creative ideas for research methods and projects. Each lesson started with a 30-minute presentation, given by one or both lecturers, followed by a 10–15 min questions-and-answers session. For the remainder of the lesson, the students engaged in various tasks within their assigned group of four, requesting assistance from the lecturers as needed.

### Data analysis

Applying the action research approach, the data collected for this study underwent qualitative thematic content analysis, with cross-referencing conducted by the authors, as a means for extracting the main recurring themes that emerged from the data. The Likert-scale items were quantitatively analyzed.

### Trustworthiness and rigor

Trustworthiness was established using a range of techniques for enhancing credibility, transferability, dependability, and confirmability, as suggested by others [e.g., (26, 27)]. *Credibility* was obtained through triangulation, a process that involves the cross-checking of the data using both qualitative and quantitative sources and methods. By incorporating multiple perspectives and approaches, we strengthened the validity of our findings. *Transferability* was obtained using rich descriptive data that offered detailed insights into the study's methodological procedures (i.e., the courses themselves). Moreover, by specifying each step of the research, we enable others to recreate this study in their own settings, thereby increasing generalizability of the results. Finally, *dependability* and *confirmability* were achieved through an audit trail, which allows readers to study the transparency of our research path. This trail of evidence helps ensure the consistency and accuracy of the study, enabling others to evaluate the reliability of our conclusions.

## Results

In this chapter, we begin by presenting the findings that emerged from analyzing the students' reflections and their free-writing feedback. Participant statements are quoted within the text as examples. We carefully chose quotes to vividly depict thoughts, emotions, or behaviors rather than providing general or vague descriptions. The principle of authenticity guided our selection of the “right” quotes, as recommended by others (28, 29). Each quote was evaluated against the following criteria: (a) it illustrates a specific point we were making about the data; (b) is concise and clearly articulated; and (c) is representative of the patterns in the data, aligning with the overall sentiments expressed by many participants. Next, we share insights from our own teacher reflections. Finally, we present the quantitative findings derived from the students' course evaluation feedback, as well as the word cloud analysis. The findings are presented by topic, as follows: (a) group work: descriptions, thoughts, and feelings; (b) mid-course reflections: changes and progress; (c) perceptions of co-teaching on the course; (d) improving teamwork; and (e) students' end-of-course evaluations.

(a) Group Work: Descriptions, Thoughts, and Feelings

The students' experiences varied, as expressed in their free-writing reflections. Some participant found working in groups to be a valuable opportunity for exchanging opinions and ideas, leading to significant progress within a relatively short timeframe. Several students also expressed their satisfaction in their ability to help others in their group, facilitating a collaborative learning environment. For example, as explained by Participant 1, “We refined each other's questions, and in the end, I think we came up with good research questions that we can start to work on;” and by Participant 28: “…the feeling that I am also helping others is exceptionally good. I feel that sharing ideas in the group helped me get to the point where I have a well-founded research question before I even turn to consult the lecturer.” Similar thoughts were expressed by Participant 17: “I really enjoyed working in a group, as I listened to the ideas of other students, from our class and from the other class, and I was also able to help them… It also helped me think of a lot of interesting ideas that I can use for my own project.”

Additional positive responses can also be seen in the following quote: “In the beginning, it was difficult for me to work with people or bond with others… Later, I was able to connect, and I really had fun working with them because they helped me a lot, and now I feel more confident” (Participant 40). The students' feedback highlighted the positive aspects of group work, demonstrating the benefits of collaborative learning and mutual support among peers. On the other hand, some students reported having encountered difficulties when working in groups. Certain students felt hesitant to expose their difficulties in front of their teammates, while others required more time to build social connections and trust with their group members, as seen in the following quotes: “Since this is the beginning of the course, we were cautious; students were initially afraid to talk about the difficulties that arose” (Participant 6); “It was difficult for me to find a research topic. A friend of mine who was in the same group helped me. But it is very difficult to get things done in a group because it takes a lot of time for everyone to think of a research topic” (Participant 19); and “Working in a group was not comfortable. We were embarrassed to help each other define a research question for the project” (Participant 13).

Following the first two lessons of the course, students described negative emotions, feeling lost, and expressing dissatisfaction, as expressed by Participant 13, who wrote: “I felt that we were wasting time, without reaching the right research topic”, and Participant 42, who wrote: “In the first lesson, I worked with someone in a pair, but it turned out that she was even more lost than I was… In the second lesson, I just worked alone.”

A few students also expressed feelings of being left behind, while other members of their group were making progress, as seen in the words of Participant 24: “It feels like we're competing with each other. As soon as someone moves one step forward, they want to continue making progress rather than taking a step back to help others. But that's really quite justified.”

When asked to elaborate on their thoughts and feelings about working in groups, some students highlighted the advantages of having others who shared similar negative experiences. For example: “Working in a group also relieves some pressure. That way, you don't feel lost, because everyone is in the same situation as you” (Participant 21); “Working with my friend is improving my self-confidence because I know that I'm not the only one who still hasn't found a research topic. It makes me feel like I'm not completely lost” (Participant 16).

Some students felt they that they need more support from the lecturer compared to others, especially those who were dealing with their first research project. Some students also found it challenging to trust their group members' feedback. When the lecturer was busy with other groups, these students felt frustrated and lost. For example, as Participant 24 wrote: “My teammates’ immaturity and inexperience prevented them from helping me move forward with my project… It feels like our discussions are not effective without the lecturer's approval… There was a feeling that the students were competing for the lecturers’ attention.”

Despite our efforts to reassure the students that their learning process was “under control,” some students felt the opposite. At the end of the second lesson, about 30% of the students had not yet decided on a research topic for their project.

(b) Mid-Course Reflections: Changes and Progress

At the end of the fifth lesson, most students felt confident that they were on the right track and began to enjoy the course. Many expressed their satisfaction with their progress on the project, even attributing some of this to their group's contribution. For example, Participant 7 wrote: “The process I went through was great. I'm satisfied with what I am doing, with the topic I chose to investigate, and with the research question that I've put together.” Similar words were expressed by Participant 4, who wrote: “I must admit that without cooperating with my teammates, it would have been very difficult for me to move forward alone,” and by Participant 16, who wrote: “I have the research topic, the relevant articles, and the desire.” In addition, Participant 21 wrote: “The course assignments extract from each person the things that they are best at… so everyone in the group brings their own contribution. It's like a jigsaw puzzle that kind of assembles itself.”

Some students described the positive change that they experienced compared to their first lessons on the course: “Compared to the first week, the situation now is good” (Participant 35); “During the first lesson of the course, I was very scared. I had no idea where to begin… Now I feel like I have the basics for conducting my research project, and I can start pouring the content into it” (Participant 17); and “My insights and confidence in the process of preparing the project are getting stronger from lesson to lesson, and I feel that this is helping me a lot” (Participant 2).

(c) Co-Teaching on the Course: Students' perceptions

Initially, some students felt more comfortable approaching the lecturer from their originally assigned course rather than the lecturer from the other course. However, as the course progressed, they learned to communicate effectively with both lecturers and with their group members. The students also began to feel more at ease asking for help and offering assistance to their teammates. As explained by Participant 15: “I had a discussion with a student from the other course, and we commented on each other's [research] questionnaire. He helped me and I helped him.” Participant 35 also said: “I sat with one of the other students in class for half an hour and really tried to help him find a research question. I felt like I was part of it.  Even [later] at home, I continued to think about the [research] question that could help him the most.” Finally, Participant 42 wrote: “I'm starting to feel really satisfied with both lecturers!!! I'm sure that I'll finish the course wholeheartedly and satisfied.”

The students also expressed their appreciation for the lecturers' support throughout the course, as seen in the following quotes: “At the beginning of the process, I was under a lot of pressure—how will I do it, when will I finish the project, etc. … Thanks to the lecturer's [positive] attitude and drive to stick to the goal, her instructional method, caring, and drive for success—I was able to get a lot done” (Participant 42); and “The lecturers are very attentive… It's very unusual and important that lecturers care so much about their students” (Participant 28). Participant 9 also wrote: “Both lecturers on the course go through every single part of a research report, they don't miss anything. They make sure that they're providing us with the necessary knowledge, just like watering a plant… Preparing the project became easier with their explanations.” Finally, Participant 21 wrote: “The lecturers’ support and piece-by-piece, step-by-step instruction throughout the course enhanced our confidence.”

From our point-of-view, as the lecturers on the course, co-teaching was a fascinating pedagogical experience for us, especially as we are both seasoned lecturers with vast experience in instructing and mentoring students on their seminar research projects. We have also worked on numerous joint research projects ourselves. As such, we were able to pool our teaching resources effectively. As written by Author 1: “I started the course with a kind of excitement and enthusiasm. That's just how I am. I like to try and experiment [with new things] and challenge myself with unique experiences, which find their way into my research and teaching… When the first lesson ended, I left the classroom with a great feeling that we are doing the right thing, something good, even.” Similar words were expressed by Author 2, who wrote: “I was full of curiosity, ready to get going.”

Managing the classroom instruction, however, proved to be more challenging than anticipated, given that the students were not accustomed to learning in groups or to co-teaching. Despite our initial doubts (it did even occur to us to revert to the original course format), we continued to encourage the students, instilling in them confidence regarding their ability to successfully complete the work. During their moments of despair, we reassured them of our commitment to their success. As expressed by Author 2: “When I first entered, the classroom was very crowded. This wasn't exactly the intimate atmosphere that I was used to from my previous research seminars… It was obvious that the students were quite surprised and didn't know what to expect.” Similarly, Author 1 wrote: “The students’ sense of helplessness was contentious and I began to wonder if we were doing the right thing. With such a short course, perhaps it's better to teach face-to-face—short and to the point. I thought of giving up and dividing the students into their original classes. But then I remembered previous situations where I had tried to initiate change. I remembered the initial confusion and uncertainty in these situations, and knew that giving up without a fight was not an option for me. So, I decided to overcome the hurdle of discomfort and move forward.”

(d) How Can the Teamwork be Improved?

When asked how they would propose improving teamwork on such courses, the students offered valuable suggestions, regarding the division of students into groups, the lecturers' instruction, and the learning environment. When writing about the *dividing of students into groups*, the students suggested: (1) re-mixing the groups every few lessons, to enhance brainstorming outcomes; (2) assigning students who are making more progress to groups with students who are facing difficulties; (3) possibly working in pairs instead of groups of four; (4) offering a list of research topics for the students to choose from, and allowing them to choose their own groups, instead of arbitrarily dividing them up; and (5) creating homogeneous work groups of students from the same original course instead of mixing students from both courses.

When writing about the instruction on the course, the students suggested: (1) scheduling a 20-min meeting each lesson for each group with one of the lecturers; (2) allocating more time for personal work during the lessons at the expense of the introductory plenary sessions; (3) adding a teaching assistant to the class to help the students (even a student who had already completed the course); and (4) determining the order and frequency in which the lecturers transition between the groups and offer advice.

Finally, when addressing the physical learning environment, the students suggested: (1) ensuring that the classroom is suitable for group work, without overcrowding or noise issues; and (2) providing options for students to work in different locations, such as the computers laboratory or the library, for those who prefer this.

(e) Students' End-of-Course Evaluations

During the final lesson of the course, and without the presence of the lecturers, the students were asked to submit their evaluation of the course and the lecturers. They were asked to rate each item on a scale of 1–7, where 1 is the lowest score and 7 is the highest. Table 1 presents the focal points of the end-of-course evaluations, as rated by the students. As seen, students wrote that they learned from the course assignments, invested a great deal of effort in the learning process, and felt that they had acquired the necessary knowledge for writing an academic seminar paper. At the end of the course students tended to prefer learning in small groups and with two lecturers, compared to the traditional teaching style.

**Table 1:** Students’ course evaluations (*n* = 40).

Items	*M*	SD
The extent of learning from the class’s final presentation	5.23	1.50
The extent of effort invested in learning on this course	6.07	0.98
The level of learning how to write an academic seminar paper	5.70	1.33
Preferred learning method for this course: traditional	4.25	2.20
Preferred learning method for this course: co-teaching	4.65	1.96

*M*, mean; SD, standard deviation.

As completing course evaluations is encouraged yet not mandatory, 10 of the 50 participants did not submit an evaluation. Thus, the responses were limited to those who were willing to take the survey.

More specific details about the students' evaluations: The data shows that the vast majority of the students felt that they had experienced significant learning during the course in general (35 students; 5–7), and from their final presentation assignment in particular (28 students; 5–7). The students also reported that they invested a lot of effort during the course (37 students; 5–7). When asked about the teaching method, on average, the students had a preference for co-teaching (4.65 vs. 4.25), although the difference is marginal. Specifically, half of the students highly rated the traditional method of teaching (20 students; 5–7), while 23 students rated the collaborative teaching. 11 students gave the maximum score to each of the teaching methods. Interestingly, a few students (less then 10) gave the same score to both methods.

The answers to the open-ended questions on the course evaluation focused on three advantages: (1) acquiring self-confidence from the learning interactions within small groups; (2) receiving thorough guidance from two lecturers; and (3) benefiting from a supportive and encouraging atmosphere that created a meaningful learning process. Among the 40 submitted evaluations, three were extremely negative, with these students expressing great disappointment and feelings of a chaotic learning environment in which they had felt lost and unable to concentrate. They wrote that they were in need of one direction to follow, but did not receive this.

Finally, as the course came to a close, the students were given the task of submitting one single word that resonated with their experience during the course. These words were then categorized into three groups: (1) positive emotions (e.g., satisfaction, good experience, support, and self-confidence) and negative feelings (e.g., frustration, difficulty, distress, and fear); (2) terms related to learning (e.g., challenge, accomplishment, enrichment, and knowledge), and (3) metaphors (e.g., tree, four, world, traffic jams, and running). Repetitions were minimal, except for five words (freedom, challenge, frustration, pressure, and support), which were seen twice.

## Discussion

Drawing from our extensive experience, conducting a research project, as required in the seminar course addressed in this study, frequently poses a formidable challenge for many of our students, with a range of difficulties that emerge throughout the process. Common hurdles involve grappling with the comprehension of English texts, composing cohesive written pieces, and becoming proficient in critical thinking. Moreover, effective time management emerges as another notable issue, as many students find it hard to allocate adequate time for fulfilling their seminar course assignments.

Traditionally, such research courses offered at our college are held in moderate sized classes, typically accommodating up to 30 students. As instructors on these courses, tasked with delivering individualized guidance and skillfully addressing the academic gaps among learners (especially in areas such as statistics and research methodologies), the act of teaching is notably demanding. Hence, our initiative to introduce alternative teaching and learning approaches stemmed from having to deal with such challenges.

While studies emphasize the importance and contribution of student engagement to their motivation, curiosity, and learning (30, 31), the literature also provides evidence as to the barriers that are entailed in creating such engagement (32, 33). In line with other research findings on cooperative learning [e.g., (34–36)], our research findings indicate that co-teaching and cooperative group learning are not an easy task—for both students and lecturers. Yet the potential for gaining deeper insights into such experiences becomes evident through such introspective research. From the students' perspective, their feedback underscored the positive aspects of group work, showcasing the advantages of mutual support and interactive learning among peers. Drawing on the symbolic interactionism theory and literature on cooperative learning, we aimed at achieving positive emotions regarding the social aspect of student-group-interactions and student-teacher interactions. Indeed, our results demonstrate that working in groups increased students' communication skills, ability to offer and ask for help, self-confidence, and enjoyment from both the learning process and the product. Yet some students expressed having encountered difficulties when participating in group activities. This could be partially attributed to their limited exposure to group work during their college studies. Predominantly, the conventional higher-education model relies on lectures being delivered by professors within traditional classroom settings, yet thereby possibly failing to equip students for successfully participating in collaborative learning scenarios (37).

In this study, the students, who are training to become teachers, were actively engaged in formulating their reflections of their experiences on the course. This endeavor therefore holds the potential to offer a better understanding of the experiences and encounters that they underwent during the course (38). The findings indicate that during the intensive yet relatively short timeframe of the course (3 weeks), several parallel processes occurred. From an *emotional* aspect, they transitioned from negative feelings of chaos and frustration to positive emotions of accomplishment and self-satisfaction. From a *social* aspect, they learned to work in groups, leveraging their strengths to help others, while learning to ask for help, listen, summarize, expand on other people's thoughts, and give and receive encouragement and support. Last but definitely not least, the students learned how to write an academic seminar paper: with great cognitive effort, they learned how to create interesting research questions, plan, read, summarize, integrate, write, and present their research—all satisfactorily achieved within the short timeframe of the summer semester.

From the lecturers' aspects, we did not encounter any personal difficulties due to our co-teaching. We were able to give each other adequate space. However, we realize that changing perceptions and behaviors takes time, for both our students and us. What we attempted was novel; we conveyed a message to our students on creativity, initiative, and innovation—not merely through words but through actions. In doing so, we conveyed that their learning is of great importance to us, and we are willing to put in the effort, think critically, implement changes, and even make mistakes.

The literature describes several co-teaching models, based on the type of interactions between the two teachers [e.g., (39)]. These models encompass a range of scenarios, including one teacher taking the lead, while the other observes or provides support (with the former baring most of the responsibility); parallel teaching of different student subgroups in the same classroom; complementary teaching that involves alternative teaching methods; and collaborative team teaching, where both educators co-teach side by side (with the responsibility being equally shared by both teachers). With knowledge of these models served as our foundation for co-teaching on this course, we made the deliberate choice of not adhering to one specific model. Instead, we remained attuned to the learners' needs, and combined different patterns of collaborative teaching based on pedagogical considerations (e.g., addressing knowledge gaps between students) and practical ones (e.g., the availability of a suitable workplace). This approach granted us the necessary flexibility for adapting the lessons and making adjustments as needed.

Implementing collaborative teaching and learning can face several limitations or barriers, some of which we have experienced as educators during this joint seminar course. These barriers can be broadly categorized into technical/administrative challenges and pedagogical challenges. Strategies are proposed to mitigate these challenges (20, 22, 40). Technical/administrative barriers include: (a) *Time constraints*: Collaborative teaching requires coordination among educators, which can be challenging due to conflicting schedules and time commitments. Solution: Establishing regular meetings and clear communication channels can facilitate alignment and effective planning; (b) *Resource allocation*: Uneven distribution of resources such as technology, classroom space, or instructional materials can hinder effective collaboration. Solution: Advocating for equitable resource allocation and seeking external funding can support collaborative initiatives; and (c) *Administrative support*: Lack of administrative support for collaborative initiatives can limit their implementation Solution: Engaging administrators early in the planning stages, demonstrating the benefits of collaboration, and seeking their commitment to providing necessary support and resources can address this challenge.

Pedagogical barriers include: (a) *Different teaching styles*: Educators may have varying approaches towards teaching, leading to potential conflicts in collaboration. Solution: Promoting a culture of respect for different teaching styles, offering professional development opportunities focused on collaborative teaching strategies, and fostering openness can mitigate this challenge; (b) *Assessment and evaluation*: Evaluating collaborative projects and attributing outcomes to individual contributions can be complex. Solution: Developing clear assessment criteria that emphasize collective achievement while recognizing individual contributions and providing training on collaborative assessment methods for educators can help address this complexity; finally, (c) *Student engagement*: Ensuring active participation and engagement from all students in collaborative activities is crucial. Solution: Using differentiated instructional strategies, assigning group roles, and incorporating peer evaluation can enhance inclusivity and accountability within groups. By addressing these limitations with proactive strategies, educators can enhance the effectiveness of collaborative teaching and learning, ultimately benefiting both students and faculty involved.

Our results underscore the advantages of teamwork for both learning and staff development. Co-teaching emerges as a powerful strategy to enhance educational experiences across diverse contexts, promoting collaboration, inclusivity, and student achievement. Looking ahead, we envision practical applications across four distinct educational contexts: learning environments, subject-specific learning, staff development, and early childhood settings.

In learning environments*,* co-teaching can enhance inclusive education in settings such as inclusion classrooms, where teachers collaborate to support diverse learners through differentiated instruction, individualized support, and inclusive practices. Additionally, in alternative education settings like alternative schools, co-teaching can personalize learning experiences, address behavioral challenges, and create a positive learning atmosphere through teamwork. Co-teaching can also be practiced virtually, in online or hybrid learning environments, where teachers collaborate to design synchronous and asynchronous lessons, provide real-time support, and engage students through multimedia tools and platforms.

Subject-specific learning can benefit from co-teaching by integrating different disciplines and promoting cross-curricular connections that deepen understanding and foster critical thinking. For example, in language acquisition programs, co-teaching enables language and content-area teachers to scaffold language development while teaching academic content, ensuring proficiency in both areas.

For staff development, co-teaching in teacher preparation programs provides aspiring educators with practical experience and mentorship, preparing them to collaborate effectively, manage classroom dynamics, and apply pedagogical theories in practice. Furthermore, co-teaching can serve as a model for on-going professional growth within professional learning communities (PLCs), where educators collaborate to refine instructional strategies and improve student outcomes based on shared best practices and data analysis.

In early childhood settings, co-teaching holds significant value, where educators can work together to create nurturing and stimulating environments that support social-emotional development and meet the developmental needs of young learners through differentiated instruction and developmentally appropriate practices.

Finally, several limitations need addressing. First, action research usually entails a cyclical process; the current research, however, only entails a single stand-alone study, with no follow up. Hence, generalizations should be made with caution. In addition, future studies could benefit from comparing the products of the course to those of traditional seminar courses, to further assess the impact of co-teaching on the quality of the academic products. In this study, due to the complexity of validating the evaluation rubric, we concentrated on measuring other aspects of co-teaching. It is therefore recommended to design a research study that compares the outcomes, and their evaluation, of courses that entail co-teaching.

### Practical implications and considerations for co-teaching

Our collaborative teaching experience in this joint seminar course enriched us professionally in several important aspects:

(1) Shared teaching materials and styles. We had the opportunity to learn about each other's teaching styles and materials (e.g., presentations, assignments, and research topics). Further discussions are needed in order to optimize the advantages of using these resources for the benefit of the students, especially in heterogeneous classes like ours.
(2) Detailed teaching plan. While we had a common understanding of the framework and content of the course, in hindsight, a more detailed teaching plan was needed. Defining the exact role of each teacher during the lessons, and allocating time accordingly, could have helped us avoid associative types of teaching, while making more efficient use of the classroom time. This may have also led to a more fruitful dialogue between us, in turn leading to common agreement regarding the priorities and importance of the topics presented in class.
(3) Flexibility and adaptation. Leaving room for flexibility and introducing changes while the course was in progress allowed us to gauge the needs of the class and adapt the lessons accordingly. Finding a balance between adhering to the pre-planned curriculum and adjusting to the actual in-class situations is even more challenging when two teachers are involved and must coordinate their actions in the classroom.
(4) Navigation between work groups. While reflecting on our teaching, we became aware of our tendency to focus on one working group for too long, sometimes neglecting the other groups in the classroom. Students who were not used to working in groups and faced difficulties with certain tasks felt lost. We now realize the importance of effectively navigating between the groups and mentoring the students while working.
(5) Shortening the plenary sessions. In our teaching practice, we often adhere to traditional methods, even during the short plenary session at the beginning of each class, where general instructions are provided for all students. Some information seemed to have been less useful for the undergraduate students at this stage of their studies. To create a more engaging learning environment, short plenary lectures of 8–10 min should be given, providing students with more time for actively working in groups.
(6) Building student confidence. During the course, it became evident that students heavily relied on our approval of their work. To boost their confidence and understanding, students should be encouraged to practice their presentation skills by giving short explanations to their peers about their research topic and its objectives. When students are confident in their work, and can effectively explain it to their teammates, they may rely less frequently on their teachers' advice.

## Conclusions

In this study, co-teaching was found to be beneficial for both the lecturers and the students. The lecturers improved their peer communication skills, including planning, instructing, and reflecting. The students conveyed increased engagement and satisfaction, as well as feelings of comfort and confidence. Our experience also shows that co-teaching and the related learning environment has a notable effect on the social interactions of students in the classroom. However, despite these advantages, developing and conducting this academic course through co-teaching required much greater resources than regular seminar courses, as seen in the additional time and effort that the lecturers invested in this course.

## Data Availability

The datasets presented in this article are not readily available because the participants filled out consent forms that the authors also filled, both agreed to anonymity. Requests to access the datasets should be directed to simaz@l-w.ac.il.
